# Functional Neurological Symptom Disorder In A Prolonged Grief Disorder Or In Depression?

**DOI:** 10.1192/j.eurpsy.2022.1175

**Published:** 2022-09-01

**Authors:** C.I. Varlam, A.M. Dumitrache, A. Itu, G. Andreea, R. Rogojină, I. Bedreagă, R. Dragomir, A. Bădescu, B. Patrichi

**Affiliations:** “Prof. Dr. Alexandru Obregia” Clinical Hospital of Psychiatry”, Section No.9, București, Romania

**Keywords:** Depression, Functional Neurological Symptom Disorder, Prolonged Grief Disorder, grief-oriented psychotherapy

## Abstract

**Introduction:**

Functional neurological symptom disorder (FND) is characterized by the ideogenic neurologic presentation deriving from unconscious stressors or conflicts. The symptoms of FND usually begin with a psychiatric illness—most commonly depression, but with the release of the latest version of International Classification of Diseases-11 (ICD-11), a new favoring factor comes to our mind: prolonged grief disorder (PGD), the newcomer to psychopathology.

**Objectives:**

The purpose of this case-report is to highlight the several key differences between PGD and depression, and the role of PGD in the onset of FND.

**Methods:**

The authors report the case of a 22 years old woman with a history of frequent seizures with loss of consciousness and the absence of stimulus-response, which started soon after the death of her 31 years old brother. Psychologically, the patient presented sustained interest in the deceased, self-blame, confusion, emptiness and low mood. On a physical exam, the patient showed periocular hyperpigmentation.

**Results:**

The emergent symptoms and signs were resistant, failed to resolve with medication alone and continued to persist across all settings. The neurological dysfunction remained present and interfered with the patient’s functioning, until applying grief-oriented psychotherapy, which was the most efficient approach.

**Conclusions:**

In conclusion, PGD represents a favoring condition for the onset of FND and it is most often mistaken with depression. Therefore, it is crucial to distinguish between these two disorders, as there is solid evidence that treatment for depression is far less helpful than targeted grief treatment.

**Disclosure:**

No significant relationships.

